# A model of tertiary lymphatic structure-related prognosis for penile squamous cell carcinoma

**DOI:** 10.1186/s12894-024-01532-6

**Published:** 2024-08-02

**Authors:** Han Tang, Zhengwei Su, Qingming Huang, Yongpeng Li, Rongchao Chen, Chengjie Ban, Chanzhen Liu, Haoyuan Lu, Xian-lin Yi, Yong Tang

**Affiliations:** 1 Department of Urology, Guangxi Medical University Cancer Hospital, Nanning, Guangxi 530021 China; 2 Department of Urology, Wuming Hospital of Guangxi Medical University, Nanning, Guangxi 530199 China; 3 Department of Gynecology and Oncology, Guangxi Medical University Cancer Hospital, Nanning, Guangxi 530021 China; 4 Department of Urology, Maternal and Child Health Hospital of Hubei Province, Tongji Medical College, Huazhong University of Science and Technology, Wuhan, 430077 China

**Keywords:** Penile squamous cell carcinoma, Tertiary lymphoid structure, Immunity, Lymphocytes

## Abstract

**Background:**

We investigated the feasibility of the tertiary lymphoid structure (TLS) as a prognostic marker for penile squamous cell carcinoma(SCC).

**Methods:**

We retrospectively collected data from 83 patients with penile squamous cell carcinoma. H&E-stained slides were reviewed for TLS density. In addition, clinical parameters were analyzed, the prognostic value of these parameters on overall survival (OS) was evaluated using ‒ Kaplan–Meier survival curves, and the prognostic value of influencing factors was evaluated using Cox multifactor design nomogram analysis.

**Result:**

BMI, T, N, and M are significant in the survival curve with or without tertiary lymphoid structure. BMI, T, N, M and TLS were used to construct a prognostic model for penile squamous cell carcinoma, and the prediction accuracy reached a consensus of 0.884(0.835–0.932), and the decision consensus reached 0.581(0.508–0.655).

**Conclusion:**

TLS may be a positive prognostic factor for penile squamous cell carcinoma, and the combination of BMI, T, N and M can better evaluate the prognosis of patients.

## Introduction

Penile malignancies are the third most common male-specific genitourinary malignancy, and squamous cell carcinoma is the most common histological type. Squamous cell carcinoma (SCC) is an epithelial malignancy that often originates from the mucosal surface of the foreskin, glans penis, and coronal sulcus, and manifests as a distal invasive or ulcerative mass. This can happen in men of any age, and risk factors include human papillomavirus, phimosis, poor foreskin hygiene, chronic inflammation such as lichen sclerosus, trauma, and smoking. Because patients with penile malignancies often delay seeking diagnosis and subsequent treatment owing to sexual organ sensitivity issues [1], there are currently no reliable markers for the diagnosis and prognosis of penile cancer.

Tertiary lymphoid structures (TLSs) are organized aggregates of immune cells formed in nonlymphoid tissue after birth [2] including typical TLSs consisting of a B-cell region containing active germinal centers and a peripheral T-cell area containing various types of dendritic cells (DCs), T cells, and hyperendothelial venules (HEVs) [3]. Currently, TLS research is focused on non-small cell lung cancer, colorectal cancer, melanoma, and breast cancer [4–9]. Several studies have shown that TLSs may be involved in antitumor immune response, and the presence of TLSs in tumors is often associated with better prognosis and clinical outcomes of immunotherapy [8]. However, the relationship between TLSs and the prognosis of penile cancer remains unclear. The purpose of our study is to clarify the relationship between TLSs and penile squamous cell carcinoma, and to provide theoretical basis for the treatment of penile cancer.

## Material method

### Study population

We retrospectively collected data from 83 patients with penile squamous cell carcinoma diagnosed with stage AJCC 2017 I and IV at Guangxi Medical University Cancer Hospital and Wuming Hospital from June 2013 to April 2023. Informed consent was obtained from the relevant research subjects, and ethical review consent was obtained from the ethics committees of Guangxi Medical University Cancer Hospital and Wuming Hospital. The patients included in the study were as follows: I, pathological diagnosis of penile squamous cell carcinoma; II. Tissue paraffin was complete, clear, and undamaged. The patients had complete medical information and follow-up records. Patients were followed from the date of treatment until death or the end of the last follow-up. Exclusion criteria: I. Patients diagnosed with penile non-squamous cell carcinoma (e.g., melanoma, urethral lymphoma), prostate adenocarcinoma, and primary bladder cancer with urethral involvement; II. Patients diagnosed with penile squamous cell carcinoma combined with prostate adenocarcinoma, primary bladder cancer, upper urinary tract urothelial carcinoma and any other malignant tumor; III. Patients with missing clinical information.

### Clinical variables

Data on the clinical features and laboratory parameters for each patient were obtained from electronic medical records. The clinical features included age, sex, AJCC stage, pathological type, recurrence, tumor volume, surgical extent, chemotherapy, and survival time. Laboratory indicators at the time of admission were collected, including APTT, PT, FDP, D-dimer, absolute neutrophil count/absolute lymphocyte count (NLR), absolute platelet count/absolute lymphocyte count [10]), CEA, CA125, CA199, SCC and Scope of operation. Scope of operation refers to the scope of surgical removal of the length of the penis, total excision refers to the operation of the penis root, body, head three parts of the total removal, part excision refers to the operation of the penis head, body part of the incision margin 0.5 cm away from the tumor.

### Pathological section

All specimens were prepared as 5 μm formalin-fixed paraffin-embedded (FFPE) sections using hematoxylin–eosin staining. After dewaxing and clarifying with xylene, the sections were dewaxed and hydrated with a series of reduced concentrations of ethanol. The slices were then soaked in hematoxylin for 3 min and eosin for 3 min. Finally, the slices were dehydrated by placing them in xylene and alcohol, and sealed with neutral resin. Each section was maintained moist [10]. All pathology sections were evaluated individually by two pathologists, with a final decision made by a senior physician.

### Calculation of tertiary lymphatic structure density

The calculated TLS density was observed with tissue FAXS cytometry at an intertumoral and peritumoral 5 mm position, labelled with an eyepiece with a 22-field of view. The TLS counts every 10X fields. Diameter (d) = 2.2 mm, S = πd^2/4 = 3.8 mm^2. TLS density = total of five random views/5/S [10].

### Statistical analysis

SPSS (version 26.0) was used for statistical analysis. Receiver operating characteristic (ROC) curve analysis was used to determine the cutoff points for TLS density, APTT, PT, FDP, D-dimer, absolute neutrophil count/absolute lymphocyte count (NLR), and absolute platelet count (PLR). The ROC curve was constructed using survival state and various indicators, and the best interception point was when the AUC was maximum. The Kaplan–Meier curves of TLS with or without stratification, surgical range with TLS, surgical range without TLS, TNM stage, recurrence, and chemotherapy were plotted, and the nomogram was designed using Cox single multivariate analysis. The consistency index (C-index) was used to evaluate the predictive ability of each factor. A value of 0.5 indicates random probability, and a value close to 1.0 indicates a better ability to correctly distinguish results. A correction curve was used to assess how well the actual results fit the nomogram model. The DCA method was used to evaluate the clinical application value of the nomogram prognostic model.

## Results

### Characteristics of patients

We retrospectively identified 83 patients with penile squamous cell carcinoma (2017AJCC I stage to stage IV), whose baseline characteristics are shown in Table 1. Among them, 57 patients had TLS in histopathological sections, and 26 patients did not have TLS. The median follow-up period was 37 months (range, 1–118), and 14 patients died in the study.

**Table 1:** Baseline data tables on clinicopathological factors and the presence or absence of TLS in patients

	Yes TLS	No TLS	*p*-value
Patient	57(100%)	26(100%)	-
Recrudescence			0.685
Yes	6(10.5%)	2(7.7%)	
No	51(89.5%)	24(92.3%)	
T123			0.900
T1	16(29.6%)	6(25%)	
T2	20(37%)	9(37.5%)	
T3	18(33.3%)	9(37.5%)	
T12/T34			0.635
T1/T2	36(63.2%)	15(57.7%)	
T3/T4	21(36.8%)	11(42.3%)	
N123/N0			0.436
N1N2N3	17(29.8%)	10(38.5%)	
N0	40(70.2%)	16(61.5%)	
N side			0.663
Single side	7(12.3%)	5(19.2%)	
Both side	10(17.5%)	5(19.2%)	
Neither side	40(70.2%)	16(61.6%)	
M			0.409
M1	2(3.6%)	2(7.6%)	
M0	55(96.4%)	24(92.4%)	
Tumor volume(cm^3^)			0.552
< 71.9	43(75.4%)	18(69.2%)	
≥ 71.9	14(24.6%)	8(30.8%)	
Corpus cavernosum penis			0.231
Yes	27(47.4%)	16(61.5%)	
No	30(52.6%)	10(38.5%)	
Corpus cavernosum urethra			0.403
Yes	34(59.6%)	18(69.2%)	
No	23(40.4%)	8(30.8%)	
Inguinal lymph nodes			0.344
Yes	16(28.1%)	10(38.5%)	
No	41(71.9%)	16(61.5%)	
Grade I/II or III/IV			0.692
I/II	39(68.4%)	15(57.7%)	
III/IV	18(31.6%)	11(42.3%)	
Scope of operation			0.726
Total excision	12(21.4%)	6(25%)	
Part excision	44(78.6%)	18(75%)	
Chemotherapy			0.941
Yes	18(31.6%)	8(30.8%)	
No	39(68.4%)	18(69.2%)	
PT(s)			0.449
< 11.85	30(52.6%)	16(61.5%)	
≥ 11.85	27(47.4%)	10(38.5%)	
APTT(s)			0.046
< 28.55	26(45.6%)	18(69.2%)	
≥ 28.55	31(54.4%)	8(30.8%)	
D-dimer(mg/L FEU)			0.034
< 0.5	33(61.1%)	8(34..8%)	
≥ 0.5	21(38.9%)	15(65.2%)	
Life cycle (month)	31(10-72)	56.5(27.0-84.0)	0.018
Squamous cell associated antigen(μg /L)	1.24(0.74-3.72)	3.8(1.31-13.75)	0.049
Cytokeratin(ng/ml)	1.96(1.31-2.46)	2.38(1.81-3.59)	0.116
Age	51.98 ± 11.2	55.15 ± 14.8	0.283
CEA	1.85(1.21-2.85)	2.16(1.39-4.72)	0.226
CA125	9.30(7.2-13.3)	10.90(7.2-17.5)	0.471
CA199	4.40(3.0-11.2)	5.35(1.35-7.59)	0.497

The data are expressed as n(%) and median (interquartile), with median (M) describing the mean of the data and interquartile (IQR) describing the degree of dispersion. TNM stage, Grade 2017AJCC penile cancer

### Kaplan‒Meier survival analysis data with and without TLS in Table 2

**Table 2:** Kaplan–Meier survival analysis table of TLS. Kaplan–Meier survival analysis table of clinicopathological factors and presence or absence of TLS

Overall Survival						
	N	Yes, TLS	*P*-value	N	No, TLS	*P*-value
TLS			0.498			
Yes	57					
No	26					
T			0.026			0.017
T1/T2	36	4		15	0	
T3/T4	21	6		11	4	
T1/ T234			0.155			0.264
T1	16	1		6	0	
T234	41	9		20	4	
N			0.031			0.088
N1/N2/N3	17	5		10	3	
N0	40	5		16	1	
N side			0.033			0.192
Single side	7	2		5	1	
Both side	10	3		5	2	
Neither side	40	5		16	1	
M			0.001			0.000
M0	2	1		2	2	
M1	55	9		24	2	
Chemotherapy			0.008			0.239
Yes	18	6		8	2	
No	39	4		18	2	
Scope of operation			0.008			0.970
Total excision	12	4		6	1	
Part excision	44	6		18	3	
Recrudescence			0.487			0.000
Yes	6	2		2	2	
No	51	8		24	2	
Tumor volume(cm^3^)			0.048			0.511
< 71.9	43	6		18	2	
> = 71.9	14	4		8	2	
Corpus cavernosum penis			0.349			0.105
Yes	27	6		16	4	
No	30	4		10	0	
Corpus cavernosum urethra			0.019			0.173
Yes	34	9		18	4	
No	23	1		8	0	
Inguinal lymph nodes			0.014			0.088
Yes	16	5		10	3	
No	41	5		16	1	
APTT(s)			0.123			0.095
< 28.55	26	7		18	4	
> = 28.55	31	3		8	0	
PLR			0.005			0.004
< 1308.3	45	4		23	2	
> = 1308.3	12	6		3	2	
NLR			0.150			0.089
< 3.06	35	4		16	1	
> = 3.06	22	6		10	3	
PT (s)			0.040			0.063
< 11.85	30	8		16	4	
> = 11.85	27	2		10	0	

*TNM stage* Grade 2017AJCC penile cancer, *NLR *Absolute neutrophil count/absolute Lymphocyte Count, *PLR *Absolute platelet count/absolute lymphocyte count

The AUC value of APTT was 0.607 (95% CI 0.4577–0.757). The AUC value for PT was 0.638 (95% CI 0.493–0.782). The AUC of tumor volume was 0.559 (95% CI 0.386–0.733). The AUC value of NLR was 0.552 (95% CI 0.372–0.732). The AUC value of the PLR was 0.753 (95% CI 0.608–0.897). In Kaplan–Meier analysis, patients with T1/T2 and T3/T4 had OS of tertiary lymphoid structure, *P* = 0.026;OS without tertiary lymphatic structure, *P* = 0.017.Patients with N0 and N + had OS of tertiary lymphatic structure, *P* = 0.031;OS without tertiary lymphatic structure, *P* = 0.088.Patients with M0 and M1 had OS of tertiary lymphoid structure, *P* = 0.001;OS without tertiary lymphatic structure, *P* = 0.000.Patients with chemotherapy and no chemotherapy had OS of tertiary lymphoid structure, *P* = 0.008;OS without tertiary lymphatic structure, *P* = 0.239.Patients with Total excision and Part excision had OS of tertiary lymphatic structure, *P* = 0.008;OS without tertiary lymphatic structure, *P* = 0.970.Patients with BMI < 21.9 and BMI ≥ 21.9 had OS of tertiary lymphoid structure, *P* = 0.021;OS without tertiary lymphoid structure, *P* = 0.423 and so on. Overall, these results suggest that BMI, T stage, N stage, M stage, lymph node status, surgical scope, presence or absence of chemotherapy, presence or absence of recurrence, and tumor volume are important prognostic factors for poor survival.

### The pathological characteristics and clinical parameters of Cox regression analysis are shown in Table 3

**Table 3:** Cox regression analysis table of TLS, BMI,T stage, N stage, M

Overall survival						
	Univariate			Multivariate		
	Hazard ratio	95%CI	*P*	Hazard ratio	95%CI	*P*
Age	1.004	0.965–1.045	0.839			
BMI	0.899	0.763–1.059	0.202	0.786	0.655–0.945	0.010
T	4.070	1.824–9.084	0.001	3.861	1.545–9.647	0.004
N	1.890	1.258–2.840	0.002	2.051	1.298–3.242	0.002
M	0.064	0.016–0.254	0.000	0.042	0.005–0.324	0.002

Multivariate Cox proportional risk analysis showed that patient OS for TLS (HR 0.077, *P* = 0.003) was an independent predictor. The OS of BMI (HR0.786, *P* = 0.010) was an independent predictor. The OS of T stage (HR3.681, *P* = 0.004) was an independent predictor. The OS of N stage (HR2.051, *P* = 0.002) was an independent predictor. The OS of M stage (HR0.042, *P* = 0.002) was an independent predictor.

**Fig. 1:**
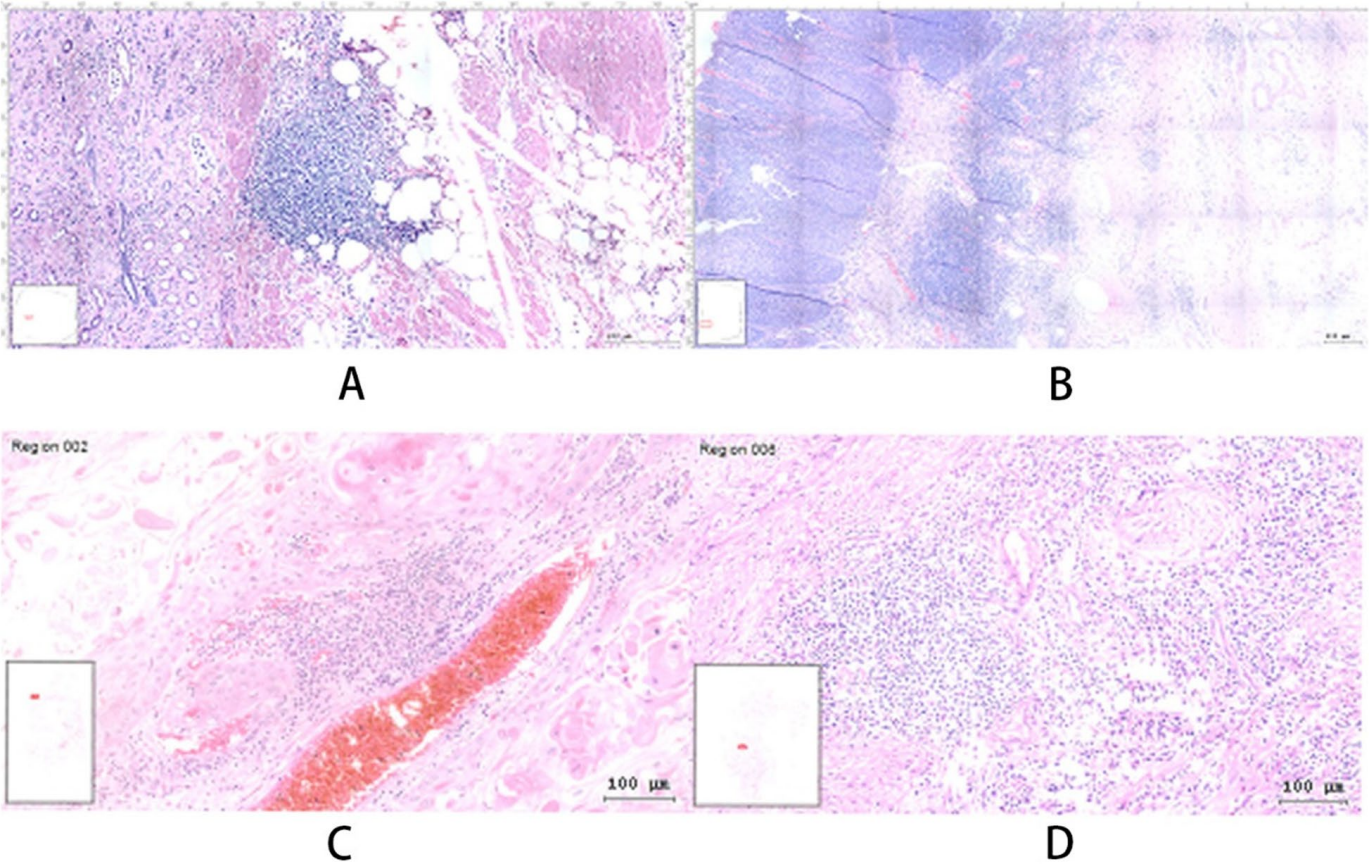
**A** and **B** show the typical tertiary lymphoid structure of two patients with penile cancer, respectively, and **C** and **D** show the lymphocyte infiltration of two patients with penile cancer

The structure of the tertiary lymphoid identified is shown in Fig. 1.

**Fig. 2:**
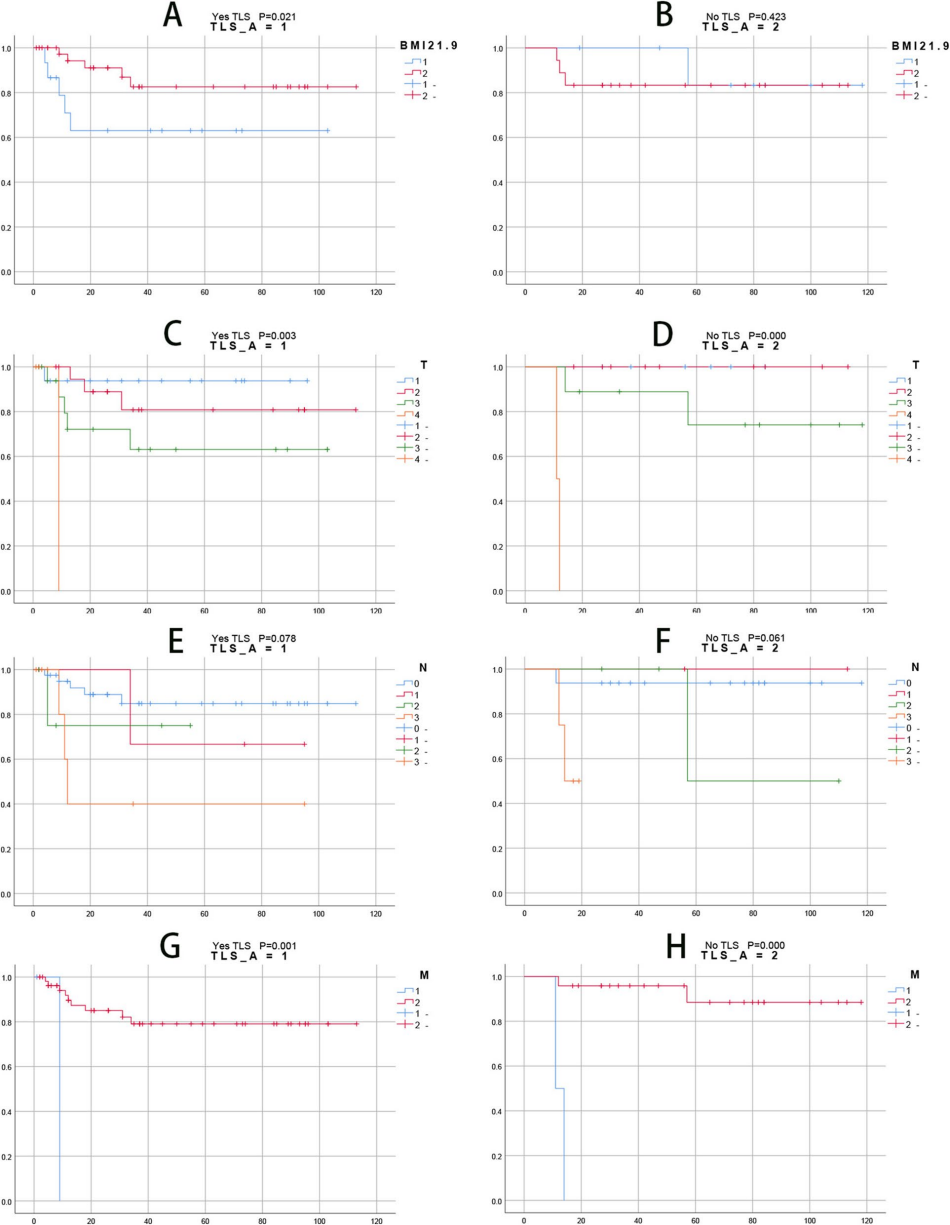
**A **shows the survival curve group of BMI21.9 with tertiary lymphoid structure, **B** shows the survival curve group of BMI21.9 without tertiary lymphoid structure, **C** shows the survival curve group of T with tertiary lymphoid structure, **D** shows the survival curve group of T without tertiary lymphoid structure, **E** shows the survival curve group of N with tertiary lymphoid structure, **F** shows the survival curve group of N without tertiary lymphoid structure, **G** shows the survival curve group of M with tertiary lymphoid structure, **H** shows the survival curve group of M without tertiary lymphoid structure

The Kaplan‒Meier survival analysis of the pathological features and clinical parameters is shown in Fig. 2.

**Fig. 3:**
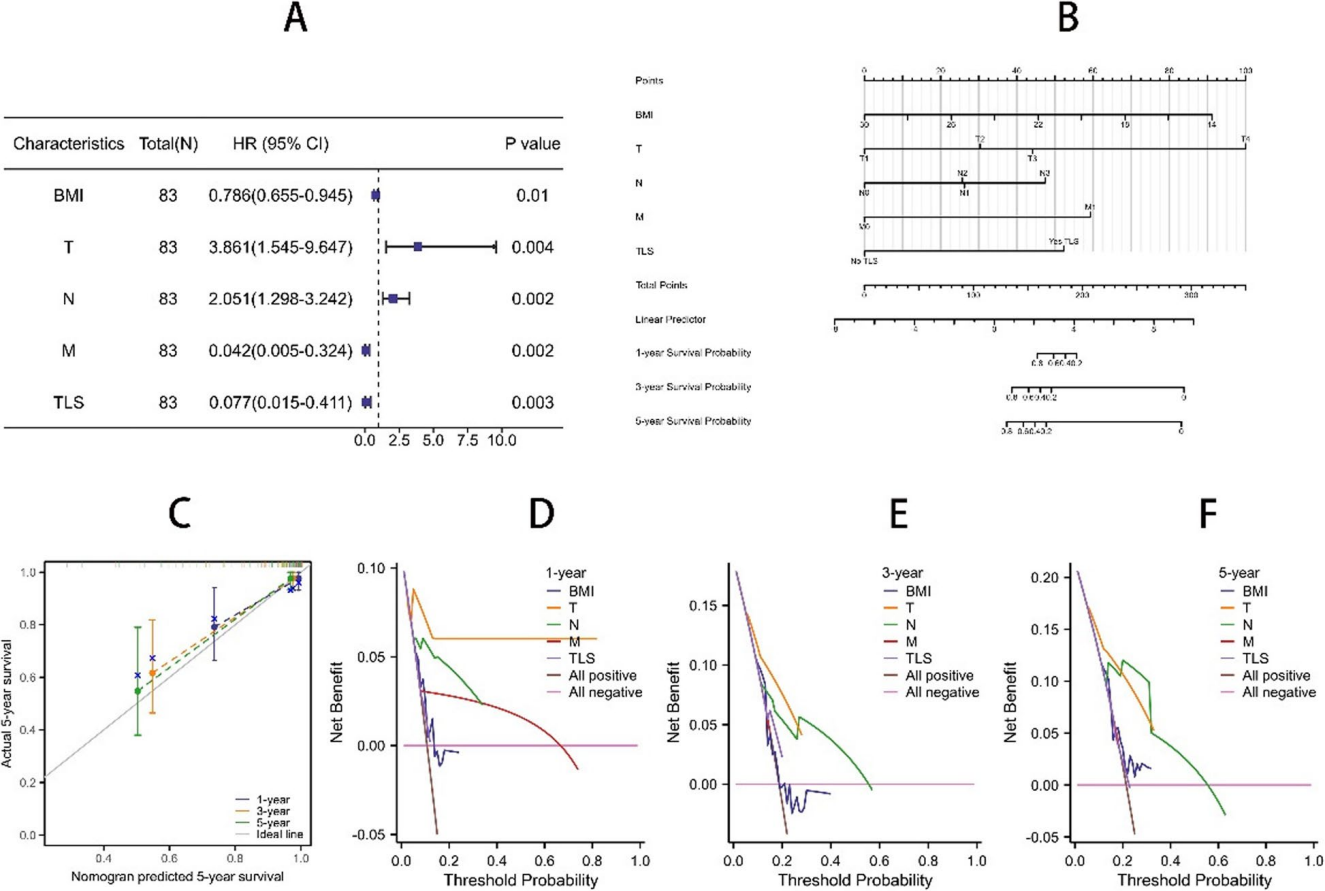
**A** is the multivariate results of BMI, T, N, M, TLS in the form of forest plots, **B** is the prediction nomogram of BMI, T, N, M, TLS, **C** is the 1-year calibration curves of BMI, T, N, M, TLS, **D** is the 1-year decision curve of BMI, T, N, M, TLS, **E** is the 3-year decision curve of BMI, T, N, M, TLS, and **F** is the 5-year decision curve of BMI, T, N, M, TLS

The pathological characteristics and clinical parameters of the patient, BMI, T, N,M, and TLS Cox regression results were used to make a forest map, as shown in Fig. 3A. The pathological characteristics, clinical parameters, BMI, T, N, and M of the patients were analyzed to make a prediction nomogram, as shown in Fig. 3B. The conformance (C-index): 0.884(0.835–0.932). The patient's pathological characteristics, clinical parameters, BMI, T, N, and M were used to make calibration curves for 1, 3, and 5 years, as shown in Fig. 3C. Pathological characteristics, clinical parameters, BMI, T, N, and M of the patients were used to make one-year decision curves, as shown in Fig. 3D.The conformance (C-index): 0.581(0.508–0.655). Pathological characteristics, clinical parameters, BMI, T, N, and M of the patients were used to make one-year decision curves, as shown in Fig. 3E. Pathological characteristics, clinical parameters, BMI, T, N, and M of the patients were used to make one-year decision curves, as shown in Fig. 3F.

### The connection between TLS and survival is shown in Fig. 4

**Fig. 4:**
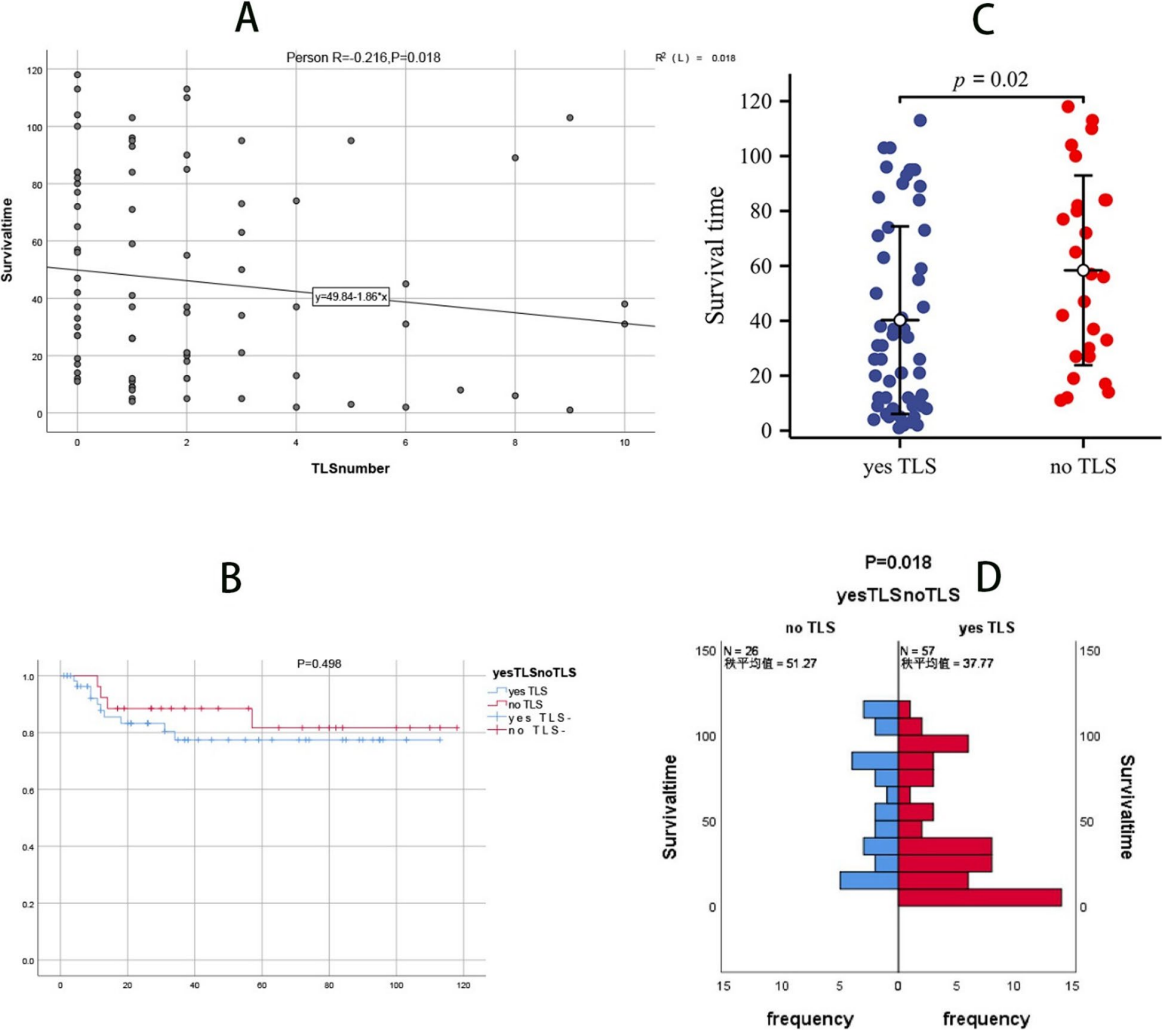
**A** is a scatter plot of time-to-life and TLS, showing the regression relationship between time-to-life and TLS. **B** shows the survival curve of 83 patients with penile squamous cell carcinoma with or without TLS expression. **C** is a comparison of the distribution between the lifetime and TLS, indicating whether there are differences in the lifetime distribution of TLS. **D** is a nonparametric test of lifetime versus TLS with or without, and there is a difference in survival time between groups with and without TLS

Figure 4A is a scatter plot of the survival time and TLS, and there is a linear regression relationship between the survival time and TLS, *R* = -0.216, *P* = 0.018. Figure 4B shows the survival curve of 83 patients with penile squamous cell carcinoma with or without TLS expression, *P* = 0.498. Figure 4C shows the distribution comparison between TLS and survival time. Whether TLS has any difference in survival time distribution, *P* = 0.02. Figure 4D is a nonparametric test of lifetime versus TLS with or without, and there is a difference in survival time between groups with and without TLS.

### TLS survival analysis in penile squamous cell carcinoma in Fig. 5

**Fig. 5:**
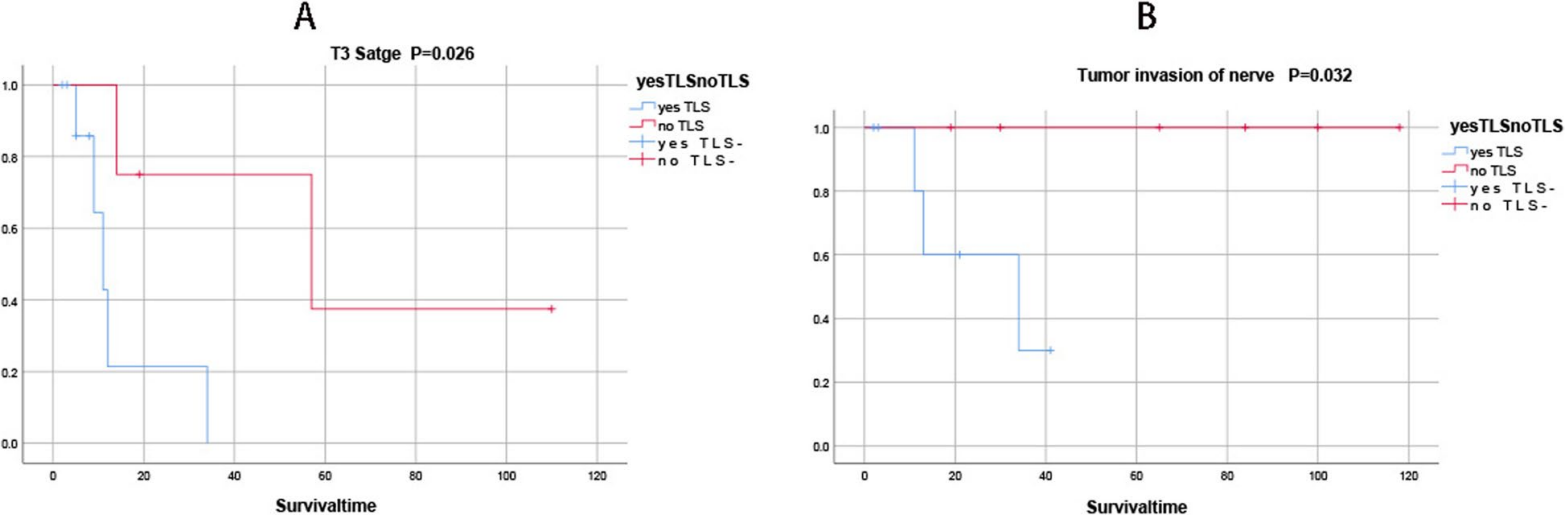
In patients with penile squamous cell carcinoma with lymph node invasion, the prognosis was better without TLS than with TLS in patients with T3 stage,*P* = 0.026 (**A**).In patients with penile squamous cell carcinoma without distant invasion, the prognosis was better without TLS than with TLS in patients with tumor invasion of the nerve, *P* = 0.032 (**B**)

With further grouping statistics, we made new discoveries. In patients with penile squamous cell carcinoma with lymph node invasion, the prognosis was better without TLS than with TLS in patients with T3 stage,*P* = 0.026 (Fig. 5A).In patients with penile squamous cell carcinoma without distant invasion, the prognosis was better without TLS than with TLS in patients with tumor invasion of the nerve, *P* = 0.032 (Fig. 5B).

## Discussion

Presence of TLS in tumors have been shown to portend a good prognosis in a variety of solid tumors [11]which may be related to the existence of sustained and effective antitumor immunity to TLSs in tumors. To the best of our knowledge, this study is the first to assess the prognostic value of TLSs in penile squamous cell carcinoma. Our results show that TLS, BMI, T stage, N stage and M stage are independent factors affecting the prognosis of patients with penile squamous cell carcinoma. Multivariate Cox regression model verifies this result again. The accuracy of TLS as a prognostic factor in clinical outcomes was further verified by decision curve and calibration curve. At the same time, the overall survival of cancer depends on TNM stage and grade. By combining TLS and TNM stage, In the future, by preventing the formation and development of TLS, we can better improve the overall survival rate of patients with penile squamous cell carcinoma.

However, due to the current treatment of penile cancer, the patients who were confined to the stage T1/T2 of penile head and did not have lymph nodes and distant metastasis could obtain better curative effect through surgical treatment. However, there were urethral cavernous invasion or accompanied by lymph node metastasis or distant metastasis, the therapeutic effect of targeted radiotherapy and chemotherapy was limited.

In Table 1, we show that there was a significant difference in the survival analysis results with and without TLSs, and many TLS studies in squamous cell carcinoma showed that TLSs were an independent prognostic factor. Tertiary lymphoid structures (TLSs) are immune aggregates with varying degrees of organization that form outside secondary lymphoid organs (SLOs) in response to chronic inflammation or infection, and TLSs are characterized by organizational patterns similar to SLOs with well-defined T-cell regions, B-cell-rich follicles, and mature dendritic cells (DCs) [12]. A higher local recurrence rate is associated with lymphatic vascular invasion and a higher tumor stage and grade [13]. Some studies have found that TLSs are more common in early cancer, which means that the formation of TLSs may start at the initial stage of tumor occurrence [6, 14, 15] and whether recurrence is more statistically significant than the analysis of the TLS group. Recurrence may be associated with a higher tumor grade, which may reduce the probability of TLS formation.

Survival time was correlated with or without TLSs, and the average survival time without TLSs was longer than that with TLSs, which may be related to the infiltration of lymphocytes around the tumor and tumor stage. In the data we collected, there were 21 T3/T4 patients with TLS and only 11 T3/T4 patients without TLS, and the higher the tumor stage, the shorter the survival time. Therefore, five of the 26 patients without TLS survived for more than 100 months, resulting in a longer average survival time without TLS than with TLS.

Squamous cell carcinoma antigen (squamous cell carcinoma antigen SCCAg) in penile cancer diagnosis research has shown that some patients with lymph node metastasis or distant metastasis can exhibit an obvious increase in SCCAg [16], namely, SCCAg is higher, the worse the prognosis, and TLS is a potential protective factor; patients with TLS have lower SCCAg.

The presence or absence of lymph node invasion, the presence or absence of inguinal lymph node invasion and the location of lymph node invasion are independent prognostic factors, and the various predictors of OS are consistent with lymph node involvement [17, 18]. To a certain extent, lymph node invasion is related to surgical scope and tumor volume, and PT is related to lymph node invasion [19].

### Limitations

Although two experienced pathologists assessed the presence or absence of TLSs and the number of TLSs in the pathological results of patients, CD3 + , CD20 + , and other markers were not used to assess the presence or absence of TLSs, so there was a certain subjectivity. Although we found that TLS, BMI, T stage, N stage and M stage showed significant differences in the survival curve with or without TLS stratigraphy, the mechanism of action remains unclear. In subsequent studies, immune markers have been used to objectively determine tertiary lymphoid structures. Further studies on the internal mechanisms are needed.

## Conclusion

TLS may be a positive prognostic factor for penile squamous cell carcinoma, and the combination with BMI, T stage, N stage and M stage can better evaluate the prognosis of patients, which may provide a new intervention direction for some patients.

## Data Availability

Datasets used and/or analyzed during the current study are available upon request to the first and corresponding author.
